# Notes From the Field: The Combined Effects of Tocilizumab and Remdesivir in a Patient With Severe COVID-19 and Cytokine Release Syndrome

**DOI:** 10.2196/27609

**Published:** 2021-05-19

**Authors:** Sabahat Ali, Sundas Khalid, Maham Afridi, Samar Akhtar, Yousef S Khader, Hashaam Akhtar

**Affiliations:** 1 Department Of Gynecology And Obstetrics Pakistan Air Force Hospital Islamabad Pakistan; 2 School of Chemical and Materials Engineering National University of Science and Technology Islamabad Pakistan; 3 Department of Biotechnology Quaid-i-Azam University Islamabad Pakistan; 4 Yusra Institute of Pharmaceutical Sciences Islamabad Pakistan; 5 Department of Community Medicine, Public Health and Family Medicine, Faculty of Medicine Jordan University of Science & Technology Irbid Jordan

**Keywords:** COVID-19, remdesivir, treatment, tocilizumab

## Abstract

SARS-CoV-2 is known to cause severe bilateral pneumonia and acute respiratory distress syndrome or COVID-19 in patients, which can be debilitating and even fatal. With no drugs or vaccines available yet, a wide range of treatment regimens used are being repurposed. The need of the hour is to analyze various currently available regimens and devise a treatment plan that is most effective for COVID-19. Here we describe the case of a 68-year-old man with hypertension and diabetes, exhibiting symptoms of cough and shortness of breath, who presented at the emergency department of our hospital. Chest computed tomography revealed bilateral ground glass opacities that were indicative of COVID-19, and a computed tomography score of 24 was indicative of severe pulmonary pneumonia. He tested positive for COVID-19. His treatment regimen included the use of convalescent plasma, oxygen therapy, steroids, high-dose antibiotics, broad-spectrum antiviral remdesivir, and anti–interleukin-6 monoclonal antibody (Tocilizumab) at various stages of the disease. Oxygen supplementation was required at the time of admission. The patient initially developed a cytokine release storm, and oxygen supplementation was initiated to manage his condition. Supportive care and multiple treatment regimens were used to successfully recover the patient’s health. With a rapid increase in number of confirmed cases worldwide, COVID-19 has become a major challenge to our health care system. With no available vaccines currently, the establishment of a combination of therapeutic drugs that effectively reduce disease progression is of utmost importance.

## Introduction

COVID-19 is a severe acute respiratory infection that has spread worldwide since the first confirmed case in Wuhan (Hubei Province, China) on December 31, 2019 [1,2]. It has since affected 235 countries, areas, or territories, with 76,900,875 confirmed cases, including 1,696,401 deaths as of December 20, 2020 [3]. The causative infectious agent for COVID-19 is a novel encapsulated, positive sense RNA virus that belongs to family Coronaviridae, and has been named SARS-CoV-2 [4]. The gold standard for COVID-19 diagnosis is a positive finding on an RT–PCR test, which confirms the presence of viral nucleic acid in blood or respiratory swab samples of a patient. Initial diagnosis of COVID-19 is based on (1) the patient’s history, which may indicate possible contact with a patient with confirmed COVID-19; (2) clinical symptoms, which range from mild or moderate (cough, fever, tiredness, or shortness of breath) to severe (pulmonary pneumonia, acute respiratory distress syndrome [ARDS]); and (3) radiological findings consistent with COVID-19 [5,6]. The first 2 cases of COVID-19 in Pakistan were reported on February 26, 2020, with 457,288 confirmed cases and 9330 deaths as on December 20, 2020 [7].

Currently available treatment alternatives for hospitalized patients with COVID-19 with severe or critical disease presentation are limited to drugs that aid in the resolution of symptoms and provide supportive care. These include the use of convalescent plasma, oxygen therapy, steroids, and broad-spectrum, high-dose antivirals and antibiotics [8]. Here we report the case of an elderly male who presented with cough, shortness of breath, and severe pulmonary pneumonia at our hospital during the early months of the pandemic. The patient was admitted after an initial examination and later tested positive for COVID-19. The patient was treated with convalescent plasma, dexamethasone, high-dose antibiotics, broad-spectrum antiviral remdesivir, and anti–interleukin (IL)-6 monoclonal antibody Tocilizumab in addition to a number of other drugs. The patient’s condition initially worsened after admission, from requiring a low flow of oxygen to developing ARDS, and required mechanical ventilation; thereafter, his condition improved, and he made full recovery and was discharged after 14 days. This case study is aimed to analyze the role of a combination of different drugs on the progression of COVID-19 in our patient and to highlight its life-saving potential.

## Case Presentation

On the evening of June 10, 2020, a 68-year-old Pakistani male physician with a history of hypertension and diabetes mellitus presented with fever, cough, and shortness of breath. Difficulty in breathing had started 3 days prior to reporting to the emergency department of our hospital and seemed to worsen with time. His vital parameters at the time of presentation included a temperature of 98.6°F, pulse rate of 85 beats per minute, blood pressure of 110/70 mmHg, and oxygen saturation (SpO_2_) of 87% in ambient air. After the initial physical examination, the on-duty physician admitted the patient to the intensive care unit for the management of possible pulmonary pneumonia and suspected COVID-19, an RT–PCR test, and chest computed tomography (CT). Chest CT revealed bilateral multifocal patchy and confluent areas of ground glass opacity (GGO) in the lungs. Associated interlobular septal thickening with an erratic paving appearance, subpleural fibrosis, and prominent lower lobe bronchi were also noted bilaterally, with more marked effects on the right side. The CT severity score was 24, which was indicative of severe disease. On admission to the intensive care unit (day 1), the patient was initially administered a one-time intravenous dose each of convalescent plasma (450 mL) and paracetamol (1 g) and administered supplemental oxygen through a non-rebreathable mask at 4 L O_2_/min to maintain an SpO_2_ of >90%. He was also started on a regimen of intravenous methylprednisolone (60 mg, once daily [OD]) and enoxaparin sodium (60 mg, OD). On Day 2, his laboratory findings indicated lymphopenia, neutrophilia, increased C reactive protein (CRP), and raised serum lactate dehydrogenase (LDH); hence, a 7-day course of two antibiotics, meropenem trihydrate (500 mg, thrice daily) and moxifloxacin (400 mg, OD), was also added to the regimen. On days 3 and 4, the patient’s condition deteriorated considerably. He was semiconscious and developed ARDS. The flow velocity of oxygen had to be increased to 15 L O_2_/min, which was still insufficient to maintain an SpO_2_ of 90%-91%. Serum ferritin levels of the patient increased, with a further increase in lymphopenia, CRP, and LDH, and as the patient developed signs of cytokine release syndrome (CRS), tocilizumab (80 mg) was administered intravenously with the dose repeated after 12 hours. On day 5, the patient was started on continuous positive airway pressure ventilation where an SpO_2_ between 88%-92% was maintained, along with a 50% fraction of inspired oxygen (FiO_2_), positive end-expiratory pressure (PEEP) of 6 cmH_2_O, and pressure support ventilation (PSV) of 14 cmH_2_O. The patient was then maintained in a prone position for up to 12 hours to manage the respiratory distress. In addition to ventilator support, dexamethasone (8 mg, twice daily [BD]) was administered intravenously. On day 8, remdesivir (200 mg) was administered intravenously with a dose halved to 100 mg for the next 4 days. A one-time dose of 20% albumin infusion (50 mL) was also administered intravenously, and a regimen of intravenous linezolid (600 mg, BD) was added. On day 10, the patient’s condition began to improve and an SpO_2_ of 97% was achieved in the same ventilator setting. Serum ferritin, CRP, and LDH levels also reverted to baseline. On day 11, the patient displayed signs of recovery, having achieved an SpO_2_ of 97%, FiO_2_ reduced to 45%, PEEP of 6 cmH_2_O, and PSV of 14 cmH_2_O. On day 12, FiO_2_ was further reduced to 40%, and the PEEP and PSV were 6 cmH_2_O and 14 cmH_2_O, respectively. The patient was advised to sit upright for 1 hour after regular intervals. Since the patient displayed signs of improvement, he was gradually weaned off the ventilator and instead provided an oxygen mask, which helped maintain an SpO_2_ of 94%-96% at 2 L O_2_/min. On day 13, patient was prescribed oral doses of dexamethasone (5 mg, OD), moxifloxacin (400 mg, OD), and linezolid (600 mg, BD) for a 3-day course. Furthermore, a reduced intravenous dose of enoxaparin sodium (40 mg, OD) for the next 7 days was advised. The RT–PCR test for COVID-19 was repeated, which yielded a negative result, and the patient was discharged on day 14 of admission (June 24, 2020).

## Discussion

### Principal Findings

This case study summarizes the clinical characteristics upon presentation, diagnosis, and treatments administered to a 68-year-old male physician with diabetes and hypertension who presented with fever, cough, and shortness of breath at our hospital. Considering his age, history (potential exposure at his work place and the presence of underlying comorbidities including hypertension and diabetes), symptoms (fever, cough, and shortness of breath), low SpO_2_ (of 87%), and chest CT findings (CT severity score of 24), our patient was suspected with COVID-19 and was at the risk of developing severe disease. A positive RT–PCR finding for COVID-19 and later stages of disease progression during the course of his hospitalization confirmed this notion; however, timely recognition of risks and the provision of immediate, effective treatment most likely ensured his recovery and survival.

In a study describing the clinical characteristics, treatments, and outcomes of 138 confirmed COVID-19 cases, Wang et al [9] reported fever (98.6%), dry cough (59.4%), and dyspnea (31.2%) as the most common symptoms associated with COVID-19. Moreover, all their patients presented bilateral GGOs on chest CT. In another study, Zhu et al [10] analyzed the chest CT scans of 72 patients with COVID-19, who were divided into two age groups: ≤60 years (n=44) and >60 years (n=32). They reported GGOs in the peripheral areas accompanied by interlobular septal thickening, subpleural line, and pleural thickening. More extensive involvement of the lobes and subpleural line and pleural thickening in the patients older than 60 years was also observed. In another study involving 51 patients with COVID-19, Li and Xia [11] evaluated whether chest CT was a reliable tool for rapid diagnosis and management of patients with COVID-19. They concluded that chest CT had a low misdiagnosis rate for COVID-19, and common features characteristic of COVID-19 include the presence of GGOs and consolidation with or without vascular enlargement, interlobular septum thickening, and air bronchogram. Furthermore, older patients showed a greater degree of lung involvement than younger patients. The chest CT findings of our patient also indicated the presence of severe bilateral pneumonia, a high CT score 24, and characteristic imaging features reportedly consistent with COVID-19 (GGO, interlobular septal thickening, and subpleural fibrosis). In addition to presenting signs and symptoms characteristic to COVID-19, where shortness of breath is indicative of severe disease, several epidemiological studies have reported that old age, male gender, and the presence of underlying comorbidities including diabetes, hypertension, cardiovascular disease, and renal and liver diseases are risk factors for severe COVID-19 [12,13]. Our patient also had some of these risk factors (old age, hypertension, and diabetes), which indicated the possibility of progression to severe COVID-19.

As many of the factors were not indicative of a possibly favorable outcome, the patient was administered a number of therapeutic interventions including convalescent plasma, anticoagulants, antibiotics, corticosteroids, immunomodulatory drugs, and antivirals during the course of hospitalization.

Based on the hospital protocol at the time, one of the first interventions our patient received was intravenous convalescent plasma therapy (CPT). CPT is being used in different countries to provide passive immunization against SARS-CoV-2. It has been hypothesized that if CPT is administered in early stages of COVID-19, it reduces the overall viral load, improves disease prognosis, and increases the chances of survival among patients with COVID-19 [14]. In a retrospective study [15], the clinical outcomes of 37 critical patients with COVID-19 who received CPT were compared with controls who were simultaneously hospitalized but did not receive CPT. Patients who received CPT displayed greater improvement in SpO_2_ and had a better survival rate.

The pathogenesis of SARS-CoV-2 and its effect on host physiology is currently under intense investigation. In cases of severe COVID-19, the progression of viral infection manifests in the form of a massive inflammatory response termed as the cytokine storm or CRS, which initially affects the lungs, causing oxygen insufficiency and leading to ARDS, but can also spread to other organs such as the heart, kidneys, and liver, leading to multiple organ failure and eventually death [16,17].

The hallmarks of CRS include high serum levels of proinflammatory cytokines and chemokines, especially the IL-1 family and IL-6, which initiate the inflammatory cascade resulting in lung inflammation, fever, and fibrosis. These changes can be monitored through laboratory parameters including the lymphocyte count, which typically decreases (ie, lymphocytopenia), and an increase in serum levels of CRP, LDH, and ferritin [18]. The presence of these circulating biomarkers is considered predictive of the severity of COVID-19 and adverse outcomes such as death among patients with COVID-19 [19]. Hence, targeting them in order to reduce the overall inflammatory cascade (ie, the CRS) is the first logical step to control disease progression [16,20].

Use of glucocorticoid therapy for the management of CRS [21] has been explored and is associated with a reduction in respiratory inflammation and improvement in lung function, often eliminating the need for invasive ventilation in cases of severe or critical COVID-19. In a study by Liu et al [22], administration of a pulse dose of methylprednisolone to 101 patients with COVID-19 resulted in improved lung function among 15 patients who were critically affected, with only 1 patient requiring mechanical ventilation. In an open-label, controlled clinical trial [23] of 2014 patients with COVID-19 who received dexamethasone and 4321 patients who received usual care, the mortality rate was markedly reduced among patients who received dexamethasone. Moreover, among patients who received dexamethasone, mortality rates in patients who required invasive and noninvasive mechanical ventilation were significantly reduced, whereas no effect was observed among patients who received no respiratory support.

In our case, the initial use of CPT and methylprednisolone was unable to curtail the cytokine storm and effectively prevent the onset of ARDS. Consequently, the patient’s condition deteriorated rapidly to the point where he was started on continuous positive airway pressure on day 5. The patient was then administered a regimen of combination therapy with 2 daily doses of dexamethasone and tocilizumab every 12 hours. Tocilizumab is an anti–IL-6 antibody preparation, which has been shown to be highly effective in improving disease severity by neutralizing IL-6, which plays a major role in inflammatory cascades and results in ARDS [24]. In a retrospective assessment of 21 critical patients with COVID-19, Xu et al [25] observed a resolution of lymphopenia in 85% of patients and a reduction in CRP levels in 84.25% of patients within 5 days of tocilizumab administration. Similarly, Guaraldi et al [26] reported that administration of tocilizumab was positively correlated with recovery and reduced the requirement of invasive mechanical ventilation at later stages.

In addition to the aforementioned immunomodulatory therapies, our patient received remdesivir on day 8. Remdesivir is a broad-spectrum antiviral agent that has previously been investigated for its inhibitory effect on viral replication in the Ebola virus, severe acute respiratory syndrome coronavirus, and Middle East respiratory syndrome coronavirus. The double-blind, randomized, placebo-controlled trial by Beigel et al [27] concluded that 541 (of a total 1062) patients who received remdesivir had a shorter recovery time (10 days as compared to 15 days in control group). Their data also suggest that remdesivir may reduce the progression of the disease to a more severe stage. Since the results of the trial were made public, remdesivir has become the first antiviral to have been authorized by the Food and Drug Association for emergency use among hospitalized adult patients at the risk of severe disease [28]. The effects of the drug are, however, closely monitored and updated with the emergence of new evidence [29]. Thus far, the preliminary results of a more recent solidarity trial led by the World Health Organization recommended against the use of remdesivir for the treatment of COVID-19 owing to its limited efficacy [30]. Moreover, in addition to ambiguity regarding its efficacy, remdesivir is also associated with acute kidney and liver injury [31].

Furthermore, it has been suggested that remdesivir may be more effective in combination with an immunomodulator such as dexamethasone or tocilizumab. A number of trials investigating the use of remdesivir in combination with tocilizumab are currently underway, and the final verdict regarding its efficacy is still pending [29,30,32,33].

In our patient, however, the combination of remdesivir, tocilizumab, and dexamethasone, administered at a favorable time (ie, during the early stages of the disease when the patient did not yet require mechanical intubation) resulted in timely resolution of the cytokine storm, resulting in an improvement in his ARDS symptoms and subsequent recovery. The underlying reason could be attributed to the effective recognition of risks and a timely administration of the available drugs.

### Conclusions

Despite being the focus of medical and scientific studies for more than a year, worldwide availability of a safe and effective COVID-19 vaccine still remains to be accomplished. Similarly, a number of antivirals and immunomodulators are being actively tested; however, an effective combination is still unavailable. Therefore, to effectively control COVID-19, there is a need to continuously explore and identify safe, effective combinations of the already available therapeutic agents. In order to ensure the safety and survival of patients, there is also a constant need to stay informed about the latest evidence. In our patient with CRS with normal liver and renal functions, combined treatment with tocilizumab, dexamethasone, and remdesivir helped reduce CRS and the subsequent requirement of mechanical ventilation. However, mixed evidence regarding the use of tocilizumab in combination with remdesivir suggests that caution should be exercised until more evidence is available for or against this combination for it to become a treatment regimen nationwide.

## Abbreviations

ARDS: acute respiratory distress syndrome
BD: twice daily
CPT: convalescent plasma therapy
CRP: C reactive protein
CRS: cytokine release syndrome
CT: computed tomography
FiO_2_: fraction of inspired oxygen
GGO: ground glass opacity
IL: interleukin
OD: once daily
PEEP: positive end-expiratory pressure
PSV: pressure support ventilation.
RT–PCR: reverse transcription–polymerase chain reaction
SpO_2_: oxygen saturation
